# The reduction of surface plasmon losses in quasi-suspended graphene

**DOI:** 10.1038/srep09837

**Published:** 2015-05-06

**Authors:** Alexander M. Dubrovkin, Jin Tao, Xue Chao Yu, Nikolay I. Zheludev, Qi Jie Wang

**Affiliations:** 1 Centre for Disruptive Photonic Technologies, Nanyang Technological University, 637371 Singapore; 2 OPTIMUS, Photonics Centre of Excellence, School of Electrical and Electronic Engineering, Nanyang Technological University, 639798 Singapore; 3 Optoelectronics Research Centre and Centre for Photonic Metamaterials, University of Southampton, SO17 1 BJ, UK

## Abstract

Highly confined surface plasmons on graphene attract substantial interest as potential information carriers for highly integrated photonic data processing circuits. However, plasmon losses remain the main obstacle for implementation of such devices. In near-field microscopic experiments performed at the wavelength of 10 μm we show that a substantial reduction of plasmon damping can be achieved by placing a nanometric polymer nano-dots spacer between the graphene layer and the supporting silicon oxide slab making graphene quasi-suspended. We argue that reduction of plasmon losses is attributed to weaker coupling with substrate phonons in the quasi-suspended graphene.

Mid-infrared graphene plasmons (GPs) have attracted tremendous interest in recent years owing to an unprecedented spatial confinement and tunability by electrostatic gating^1^,^2^,^3^,^4^,^5^,^6^,^7^,^8^,^9^ which are the key features for building the next generation photonic and optoelectronic devices^10^,^11^,^12^,^13^. Two dimensional structures of graphene lead to high charge carrier mobility^14^,^15^ that potentially promises GPs with a low loss and a large propagating distance^7^. Theoretically, this is realized in suspended graphene at mid-infrared frequencies, below the intrinsic optical phonon branch (ħω < ħω_Oph_ ≈ 0.2 eV) and sufficiently high doping level (ħω < ħω_inter_)^7^. However, the mobility in supported graphene strongly depends on the properties of the substrate/environment surrounding the graphene; this has a direct effect on the performance of graphene devices^16^,^17^,^18^,^19^,^20^. Recently, mid-infrared spectroscopic studies uncovered the crucial role of substrate optical phonons in damping of GPs^21^,^22^. To date, the direct experimental mapping of propagating mid-infrared GPs^23^, as well as the scanning plasmon interferometry^1^,^2^,^24^ have demonstrated only a very short propagation distances (about several plasmonic wavelengths). Therefore, further experimental study of GPs damping mechanisms and approaches to increase the propagating distance are highly important for the development of future on-chip mid-infrared plasmonic devices^13^,^21^,^25^,^26^,^27^.

GPs damping due to the coupling with the substrate optical phonons can be reduced by shifting an excitation wavelength out of the corresponding phonon line, as has been demonstrated^22^,^23^. While only few studies discuss the intriguing mechanisms of interaction between mid-infrared GPs and optical phonons in thin films^28^,^29^,^30^. Study of GPs damping control by an ultrathin film or nanometric spacer as comparable to the thickness of monolayer graphene, inserted between a bulky substrate and the graphene would be of high interest, and it may open up a new pathway for the damping reduction and control in graphene plasmonic devices. Experimentally, it is more effective to study such control of damping/coupling between bulky phonons and GPs, in the case with strong substrate-graphene interaction.

Additionally, as it follows from theoretical analysis^7^, relaxation time in sufficiently doped graphene, which represents GPs damping, is proportional to DC charge carrier mobility at mid-infrared frequencies. Thus the loss of mid-infrared GPs is affected not only by direct optical coupling with substrate phonons, but also depends on specific properties of the graphene environment/substrates, as this affects the mobility of graphene. DC mobility in graphene is mainly degraded due to carrier scattering on (1) charge impurities and (2) remote phonons^31^,^32^,^33^,^34^. The first factor may appear as a result of absorbents attracted to the substrate^35^ or owing to the intrinsic properties of the substrate^36^, while the second factor appearing at polarizable substrates such as SiO_2_ and SiC^34^ exponentially depends on the substrate-graphene separation distance^37^. Since the suspended graphene isolated from the substrate has demonstrated high mobilities, a possible device which utilizes a suspended or partially suspended graphene may benefit from suppressing the damping of mid-infrared GPs.

In this work we experimentally study the effect of quasi-suspended graphene on mid-infrared GPs. The monolayer graphene is placed above a chemically engineered nanostructured spacer (NS) on the substrate, that leads to a “quasi-free-standing”-like graphene^38^,^39^ structure used for our plasmonic damping measurements. By direct mapping of GPs with scattering-type scanning near-field optical microscope (s-SNOM)^40^,^41^ we uncover one possible pathway to reduce the damping of mid-infrared plasmons in graphene. Finally, we compare our experimental results with numerical simulations based on a developed numerical model.

## Results and Discussion

**Figure 1:**
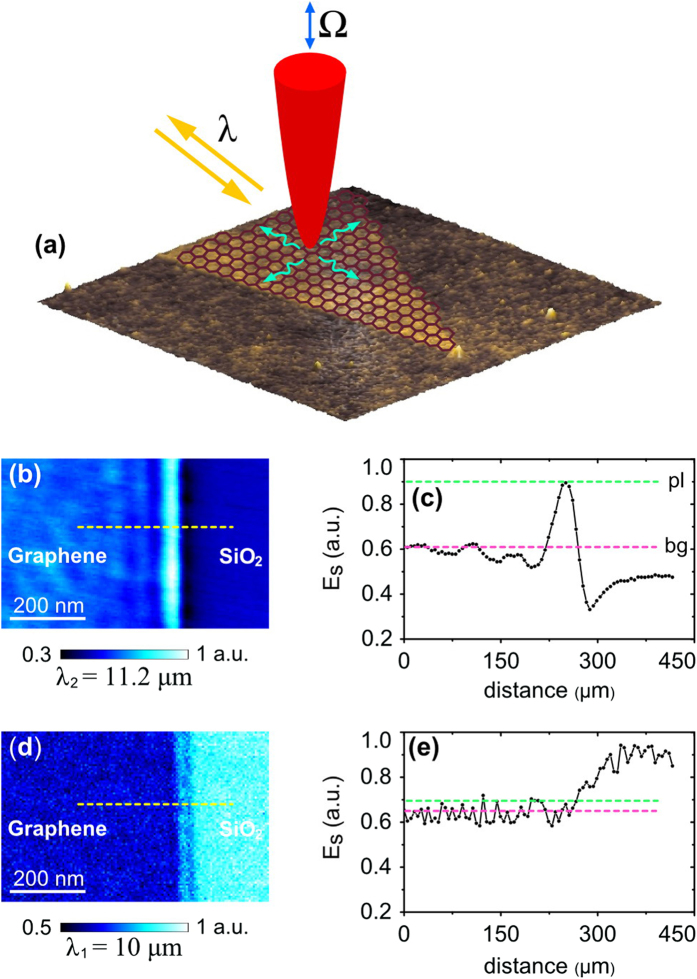
Illustration of the damping of graphene plasmons on silicon dioxide substrate at mid-infrared frequencies (**a**) A sketch of the principle behind scattering-type scanning near-field optical microscope (s-SNOM) measurements. (**b**,**d**) Optical near-field images of exfoliated graphene flakes, recorded at λ_2_ = 11.2 μm and λ_1_ = 10 μm, respectively. (**c**,**e**) Corresponding cross-sections along dotted yellow lines in images (**b**,**d**) which represent a distribution of the near-field across graphene-SiO_2_ boundary. Green and pink dotted lines mark GPs’ field magnitudes at the first maximum close to the boundary ((Es)_pl_), and inside the inner part of graphene flakes (background value, (Es)_bg_).

To experimentally image GPs, we use scanning plasmon interferometry technique^24^,^42^ which was used in the pioneering works revealing the mid-infrared propagating plasmons in graphene^1^,^2^. The technique utilizes a sharp metalized tip to overcome the large plasmon-photon momentum mismatch typical for GPs^12^. The tip strongly confines incident light and launches propagating plasmons in graphene (see sketch in Fig. 1a). In the experiment, the tip is illuminated by either a quantum cascade or carbon dioxide laser which provide a wavelength of λ_1_ = 10 μm or λ_2_ = 11.2 μm correspondingly. A p-polarized beam is focused on the tip. This generates, in each point during the scanning, cylindrical GPs travelling in all directions out of the tip along the graphene flake. Launched GPs are reflected back from the graphene edges, collected by the tip and scattered out in the far-field. Pseudo-heterodyne interferometric detection of the scattered light allows us to record the amplitude of tip-scattered field, Es, as well as topography in each point of the scan (see Methods section for more details).

We investigate on samples which initially (when no spacer is applied) possess strongly damped GPs. Experimentally this is realized at an excitation wavelength λ_1_ = 10 μm due to the strong coupling between GPs and SiO_2_ optical phonons^22^,^23^. All graphene samples are fabricated by mechanical exfoliation of highly ordered pyrolytic graphite (SPI suppliers) with a Scotch tape. To define a factor which represents the damping of GPs we first compare near-field images of GPs with a different damping strength. Practically, as an example, this comparison is done on the same graphene sample, measured for two different excitation wavelengths, in and out of the substrate phonon line, respectively. Fig. 1b,d shows two typical near-field images of the edge of a graphene flake fabricated on a clean 285-nm thermally oxidized Si-SiO_2_ wafer, taken at λ_1_ = 10 μm and λ_2_ = 11.2 μm. Corresponding cross-sections of Es along yellow dotted lines are plotted in Fig. 1c,e. While at λ_2_ = 11.2 μm, GPs appear as half GP-wavelength spaced fringes, at λ_1_ = 10 μm plasmon field is significantly damped and nearly invisible due to the strong interaction with substrate phonons. In this paper, we use the ratio R between the first maximum of the electric field (close to graphene edge) and the field magnitude in the inner part of the flake, (Es)_pl_/(Es)_bg_, to represent the damping rate of GPs. Moderately damped GPs at λ_2_ = 11.2 μm (out of SiO_2_ phonon line) is characterized by R ≈ 1.5, while for λ_1_ = 10 μm, the ratio R ≈ 1. Thus, different damping values are characterized by different R. In the following study we will experimentally analyze a possible enhancement of the parameter R for the case of excitation at λ_1_, by inserting a spacing layer between graphene and the SiO_2_ substrate.

To produce graphene-SiO_2_ spacer we apply the following fabrication processes: (1) thermally oxidized Si-SiO_2_ wafer is covered with a photoresist by spin-coating, followed by softbaking (2) the photoresist is partially cross-linked by dry-etching, (3) the sample is sonicated in a stripper and rinsed with 2-propanol (see Methods section for more details). This leads to the formation of few-nm thick nano-dots layer of novolac-based polymer^43^ on the wafer, which we use for lifting and partial suspension of graphene above silicon dioxide. Separately, we have also checked that sonication process of the clean wafer in the stripper does not visibly affect the SiO_2_ roughness and the damping-related factor R in the graphene (Supplementary Information, section S1).

**Figure 2:**
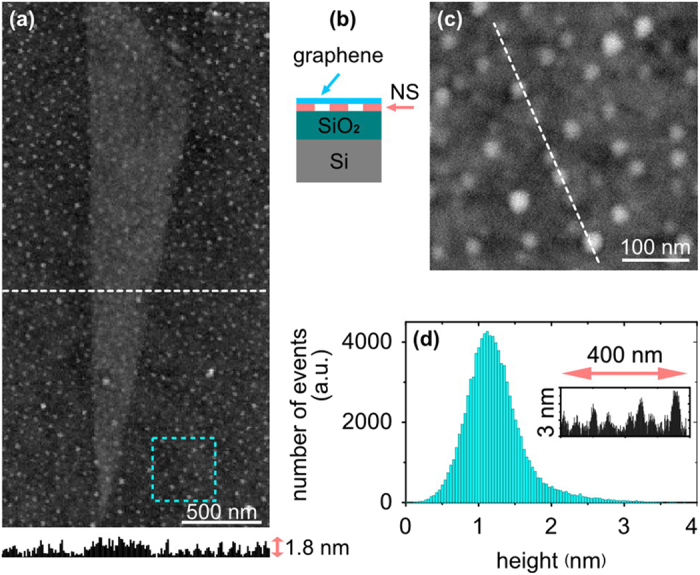
Topography of the graphene flake, exfoliated on top of NS-SiO_2_ (**a**) Atomic force microscopy (AFM) image and a corresponding height profile along the dotted white line. (**b**) Side-view schematic of the graphene on top of NS-SiO_2_. (**c**) Zoomed-in AFM topography of the area inside the dotted cyan square in image (**a**). (**d**) Height histogram of the data in the image (**c**); inset shows a height profile along the white dotted line in the image (**c**).

Graphene is then mechanically exfoliated on top of the fabricated spacer. A typical atomic-force microscope (AFM) image and the height profile of a graphene flake above NS are shown in Fig. 2a. The tapered-ribbon shape of the flake of graphene is clearly defined in the middle of the image. Graphene appears as a sheet with a thickness less than 1 nm, partially conformed to the engineered nano-dots. We proof that graphene exfoliation takes place in a monolayer by Raman spectroscopy (Supplementary Information, section S2). Additional measurements and statistical analysis show that a roughness (rms) and an average relative height of the graphene-on-NS is reduced in comparison with bare NS surface (Supplementary Information, section S3). This is a direct experimental evidence of gaps between SiO_2_ and graphene sheets. From the other hand, the recorded topography may display a larger degree of conformation of the graphene to NS (and, correspondingly, less efficient suspension) than it actually presents in the sample. It is well-known that AFM records the tip trajectory as it goes along the surface, rather than the true topography of the sample during measurements^44^. Thus, the visible conformation may not represent a true topography of the flake since the suspended monolayers can be easily bended at a nanometer scale under the mechanical force from AFM tip^44^,^45^,^46^. This nm-scale bending happens only in the current point of the scan, therefore does not affect the mobility and plasmons propagation along the whole flake. Since graphene is placed on the nanostructured surface, it is partially suspended in gap areas between the nano-dots^47^.

Additional scratching and AFM measurements of NS close to the flake location, as well as presented further optical s-SNOM data, show that nano-dots have been grown directly on SiO_2_ without any continuous base-layer of polymer beneath. Zoomed-in AFM topography inside the cyan dotted square in Fig. 2a is presented in Fig. 2c. The corresponding height histogram (Fig. 2d) and cross-sections analysis (inset in Fig. 2d) show that fabricated NS on top of SiO_2_ surface is formed by randomly placed nano-dots with an average height of 1.2 nm, a typical size of 20 – 40 nm and a typical spacing of 10 – 100 nm.

**Figure 3:**
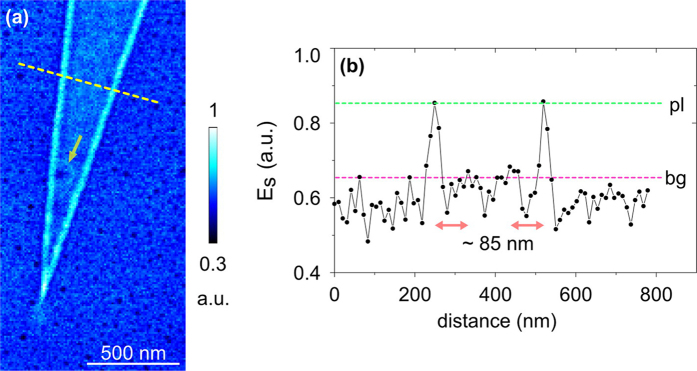
Near-field imaging of the graphene on NS-SiO_2_, performed at λ_1_ = 10 μm (**a**) Near-field optical image. The yellow arrow marks an optical ring structure around a single “extra-high” NS dot beneath graphene. (**b**) Corresponding cross-section along the dotted yellow line in image (**a**) Green and pink dotted lines mark GP field magnitudes at the first maximum close to graphene edges, and inside the inner part of the graphene flake. Red arrows highlight a distance between first and second maxima of GP field distribution.

In Fig. 3a we plot a typical optical near-field image of graphene supported by engineered NS on SiO_2_ (at λ_1_ = 10 μm). The image corresponds to the bottom part of the tapered ribbon displayed in Fig. 2a. As can be seen from data the electric field is concentrated close to the edges of NS-supported graphene flake in a much stronger fashion than in the case of bare SiO_2_ substrate (Fig. 1d,e). The ratio R, estimated from the corresponding cross-section (graph in Fig. 3b), reaches the value of about 1.3. The tip of the graphene ribbon taper localizes plasmon fields stronger than graphene edges that are distant from the tip part. These observations agree with the previously reported typical pictures of mid-infrared GPs^2^ for the case of moderate damping. This directly support the benefit of NS as an efficient approach for the reduction in damping. Nanostructured spacer increases corresponding visibility of GPs in s-SNOM from a crucially-damped level (Fig. 1d,e) to a moderately-damped and visible case (Fig. 3). We further verify it through near-field measurements of more than 10 different flakes fabricated above NS. Several additional images are presented in the Supplementary Information, section S4.

A typical feature of the near-field images of NS-supported graphene is the lack of polymer dot optical fingerprints in the field distribution inside the flake areas. The substrate part (Fig. 3a) appears as a uniform background (SiO_2_ signal) with randomly distributed black spots which represent the near-field signal of polymer dots. Therefore, we conclude that the roughness in topography observed within NS beneath the flake does not lead to considerable reflections/scattering of GPs field. This agrees with previously reported observations^38^ of GPs reflections at nanometer-size steps in quasi-free-standing graphene on SiC, where authors derived a critical step-height of about 1.5 nm below which, no reflection appears. In our observations, as it is seen from Fig. 2, the majority of the polymer dots have a height within the 1.5 nm range. As an exception, marked with a yellow arrow in the Fig. 3a we observe the reflection of GPs around an “extra-high” polymer dot with a height of about 3.5 nm. The field distribution around the dot features a center minimum and surrounding bright ring structure. A distance ∆ between the center of the minimum and the ring is about 75 nm. We suggest that the estimated ∆ is related to the half wavelength (λ_gp_/2) of propagating GPs reflected from the dot. Additionally, as it can be seen from Fig. 3b, the optical field profile features two faint secondary maxima at the distance of about 85 nm from first maxima at both sides of the flake, that we ascribe to the plasmon interferometry. All mentioned regularities are a typical attribute related to propagating mid-infrared GPs, thus demonstrating the beneficial effect of employing NS for damping reduction at λ_1_ = 10 μm. Additional, study of the same graphene flake at λ_2_ = 11.2 μm, where SiO_2_ phonon damping effects can be excluded (Supplementary Information, section S5), shows propagating plasmon interferometry pattern with the damping comparable to graphene on bare SiO_2_ (Fig. 1b,c and Supplementary Fig. S1c,d). Considering no visible improvement of the interferometry pattern at λ_2_ = 11.2 μm, we assume that the carrier mobility in graphene on NS compare to graphene on bare SiO_2_ is most likely, slightly degraded due to the scattering at nano-dots of the spacer.

**Figure 4:**
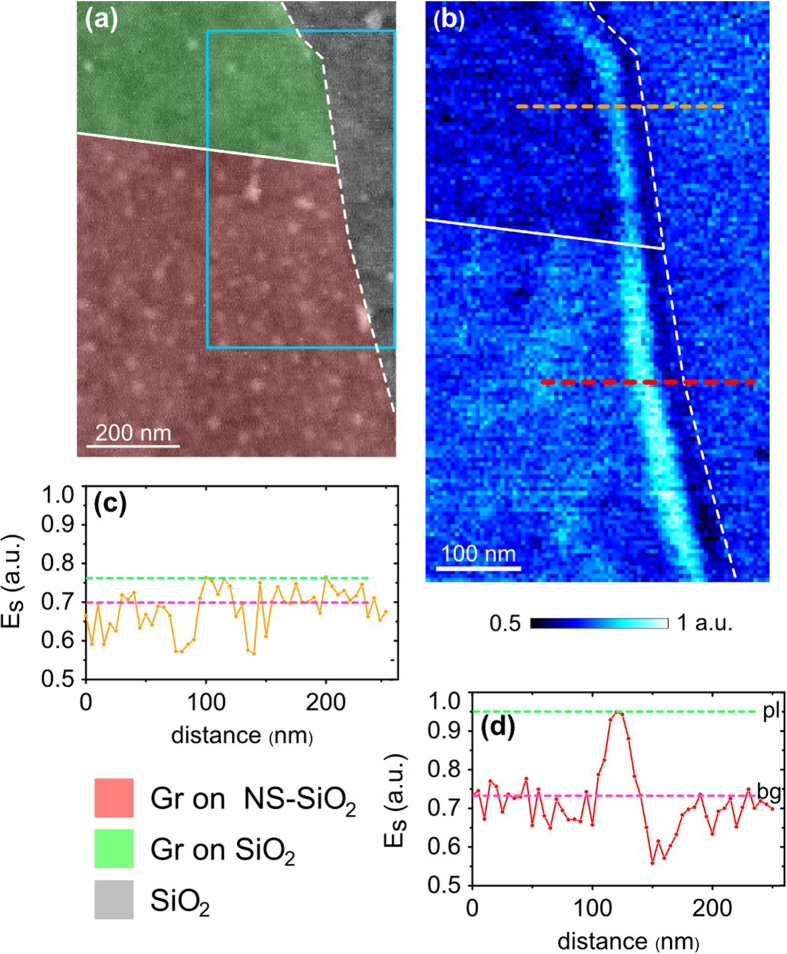
Demonstration of GPs damping suppression in graphene, exfoliated directly at the boundary between SiO_2_ and NS-SiO_2_ (**a**) AFM topography; the pink and green colours highlight graphene on NS-SiO_2_ and graphene on approximately clean SiO_2_, respectively. (**b**) Near-field optical image (recorded at λ_1_ = 10 μm) of the graphene flake, corresponding to the region marked with a cyan frame in image (**a**) solid white line highlights the boundary between graphene on NS-SiO_2_ and graphene on SiO_2_; dotted white line marks the edge of the flake. (**c,d**) Corresponding cross-sections along orange and red dotted lines in image (**b**); green and pink dotted lines in images (**c,d**) mark GP field magnitudes at the first maximum close to the graphene edge, and inside the inner part of the flake.

To further analyse the effects of NS on GPs, we study a single exfoliated graphene flake which is located directly on the boundary of NS and almost clean SiO_2_ regions. Fig. 4a displays topography of the sample, where graphene on NS and graphene on SiO_2_ are marked with pink and green colours correspondingly. As it can be seen from the data, the green area contains only several nano-dots, therefore can be considered, approximately, as bare SiO_2_; while the pink region is covered with well-developed nanostructured layer. Grey part represents the SiO_2_ substrate, which is not covered with graphene. Dotted white line marks the edge of the graphene flake. Solid white line highlights the boundary between graphene-NS and graphene-SiO_2_ areas. An optical near-field image (Fig. 4b) of this boundary has been recorded for the region marked with a cyan rectangle in Fig. 4a. On the boundary the flake is divided into two regions: with a higher and a lower near-field scattered signal strength. Electric field distribution close to the edge of the flake has a typical maximum with a magnitude switching while passing across the boundary. Field cross-sections, Es, along dotted orange and red lines are plotted in Fig. 4c,d correspondingly, from where the values of the ratio R have been estimated. It is important to mention that not only the field magnitude, but also the damping-related factor R increases along with passing across the boundary transition. The value R in graphene-SiO_2_ area is about 1.1, while in graphene-NS region it reaches the value of more than 1.3, thus directly demonstrating GPs damping decrease, in the same graphene flake as a result of NS implementation.

**Figure 5:**
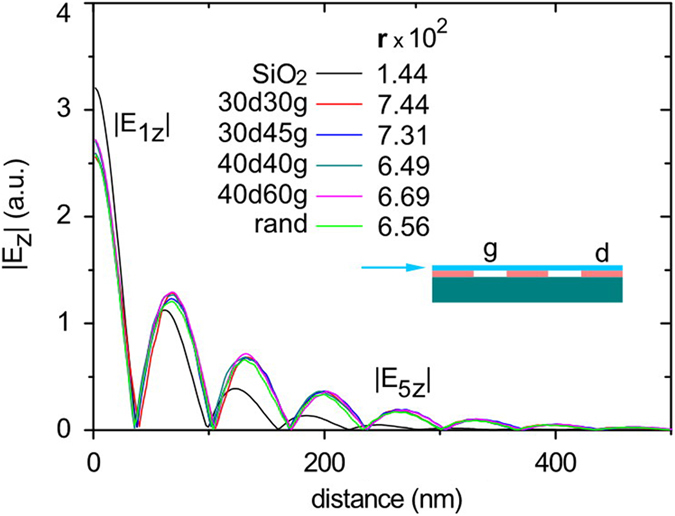
>Numerical simulations of GPs propagation and damping on NS-SiO_2_ at λ_1_ = 10 μm Inset shows a sketch of simplified 2-D model of the spacer; where d is the nano-dot size, g is the gap between neighbouring dots, and blue arrow represents the direction of the plasmon launching. The data marked with light green colour was calculated for the random geometry of the spacer (from the left to the right: d_1_ = 40 nm, g_1_ = 45 nm, d_2_ = 60 nm, g_2_ = 40 nm, d_3_ = 45 nm, g_3_ = 60 nm, d_4_ = 30 nm, g_4_ = 30 nm, d_5_ = 45 nm, g_5_ = 60 nm, d_6_ = 45 nm). Left-hand side axis displays an absolute value of the vertical component of electric field, taken at 3 nm distance above graphene. The damping strength r, which is defined here as a ratio between fifth and first maxima of the field (r = |E_5z_|/|E_1z_|), is given for each of calculated NS geometries in the inset of the figure.

Finally, the physics of mid-infrared plasmon damping and propagation in graphene on NS-SiO_2_ at λ_1_ = 10 μm is treated by numerical simulations based on the finite-difference time domain method. To describe spacing and partial suspension effects on GPs, we implement a simplified two-dimensional model as schematically displayed in the inset of Fig. 5. The optical conductivity of a graphene sheet is calculated in the random phase approximation^26^,^48^. The thickness of the polymer spacer is set as 1.2 nm, based on the average experimental value. The Fermi energy and mobility of graphene are taken as: E_f_ = 0.4 eV and μ = 10000 cm^2^/(V∙s); the refraction index of the polymer n = 1.45. The GP is launched in the graphene from the left-hand side. The evolution with the distance of the absolute value of vertical component of plasmonic electric field is plotted in the graph in Fig. 5. We characterize the damping rate of GPs by a ratio (r) between the fifth and the first maxima of the field, which is presented for several spacer parameters (see inset in Fig. 5). This data shows an increase of this parameter by about 4-5 times for all calculated NS geometries, compared to graphene on bare SiO_2_, that represents a significant suppression of damping and agrees with the experiment. Our model describes only the effect of spacing and partial suspension of graphene sheet from SiO_2_, while the value of the mobility is fixed.

In summary, we demonstrate that lifting exfoliated graphene from the silicon dioxide surface with an ultra-thin nanostructured polymer spacer, helps with the control of mid-infrared plasmon damping and propagation. Polymer nano-dots result in spacing and partial suspension of graphene that is beneficial for remote phonons screening. Owing to the ultra-small thickness of the spacer, the nanoscale roughness does not lead to strong chaotic reflections of GPs at the polymer nano-dots, that is one of requirements for unperturbed performance of possible devices fabricated in graphene on top of NS. Numerical simulations of plasmons propagation in graphene placed above silicon dioxide covered with polymer nano-dots show an increase of the propagation length and a suppression of damping that is in agreement with the experiment. This work contributes to understanding of mid-infrared GPs damping mechanism, and gives insight into the fundamental problems of interaction of the plasmons with deeply subwavelength nanostructures.

## Methods

### Fabrication of the nanostructured spacer

To fabricate the NS, we use the polymer which is, essentially, self-organized as a highly nanostructured film during the process of ultrathin (nanometric) films fabrication. The following process was performed. (1) Virgin thermally oxidized Si-SiO_2_ wafer (285 nm oxide thickness) was covered with AZ 5214E photoresist by spin-coating at 4000 rpm for 30 s. The thickness of the resulting photoresist film is about 1.6 μm. (2) The wafer was then softbaked for 100 s at 105 °C. (3) The resulting sample was dry-etched for 4.5 min in the Plasma-Therm 790 series RIE (CF4 etchant, pressure 80 mtorr, RF power 100 W). The dry-etching process leads to a higher degree of cross-linking, within the novolac-based polymer component of the photoresist due to a possible overheating/UV exposure from the plasma. The dry-etching does not remove the photoresist film completely with the chosen etching time. (4) Then the sample was sonicated in NI555 stripper for 5 min at room temperature, and finally, it was rinsed thoroughly with 2-propanol and dried with nitrogen.

### Near-field microscopy

Our setup is based on a commercial s-SNOM (Neaspec GmbH) performing measurements with a Pt-coated Si tip at the tapping frequency Ω of about 250 kHz and the tapping amplitude of about 60 nm. We use Daylight Solutions tuneable QCL laser and temperature-tuneable Access Laser CO_2_ laser. Background-free near-field imaging is given by the demodulation of recorded optical signal at higher harmonics of the tapping frequency (in this work we use the 4^th^ harmonic signal). We note that, as commonly known, near-field optical images recorded with the QCL laser (λ_1_ = 10 μm) typically show lower signal to noise ratio compare to the CO_2_ laser (λ_2_ = 11.2 μm). With reference to Neaspec GmbH, this difference in performance is mainly attributed to intrinsic laser specific aspects in the sense how the mid-infrared lasing is realized in the two lasers.

**Numerical simulations** are based on the finite-difference time domain method presented in the reference^26^. To study GPs propagation in quasi-suspended graphene, we use a simplified two-dimensional model of graphene seating on the nano-dots, where nanodots sizes and separation distances are referred to experimental values. In simulation we characterize the damping strength of GPs by direct comparison of the field magnitude after certain plasmon wavelengths of propagation (in this work we define this damping factor as a ratio between the fifth and first maxima of the field). This ratio naturally quantitatively represents the field damping along the GPs propagation direction in graphene. In experimental measurements at λ_1_ = 10 μm we can define the first field maxima close to the graphene edge and the field magnitude in the inner part of the flake, while due to strong damping, it is not possible to clearly define multiple maximums of GPs field over the flake. Therefore we implemented another damping-related factor, (Es)_pl_/(Es)_bg_, which compares the near-field magnitude in the first maximum and the inner part of the flake where a plasmon, reflected from the edge, has been already completely damped. As it was verified above, on the example of strong and weak damping, this factor can be used for qualitative comparison of GPs damping strength.

## Author Contributions

A.M.D. proposed the idea of the paper, performed samples fabrication and near-filed measurements, analysed data and prepared the main manuscript text. J.T. performed numerical simulations. J.T. and X.C.Y. contributed to samples fabrication. Q.J.W. and N.I.Z. supervised the project and contributed to the manuscript text. All authors discussed the results and commented on the manuscript.

## Additional Information

**How to cite this article**: Dubrovkin, A. M. *et al*. The reduction of surface plasmon losses in quasi-suspended graphene. *Sci. Rep.* **5**, 09837; doi: 10.1038/srep09837 (2015).

## Supplementary Material

Supporting Information Supplementary Figure S1-S5
