# Supplementary material for: The reduction of surface plasmon losses in quasi-suspended graphene
Source: Sci Rep. 2015 May 6;5:9837. doi: 10.1038/srep09837 (PMC4421827; doi:10.1038/srep09837)
Supplement: Supporting Information — Supplementary Figure S1-S5 [file srep09837-s1.pdf]

Supplementary Information

The reduction of surface plasmon losses in quasi-suspended graphene

Alexander M. Dubrovkin[*,†], Jin Tao[†,‡], Xue Chao Yu[‡], Nikolay I. Zheludev[†,§], Qi Jie Wang[*,†,‡]

[†]Centre for Disruptive Photonic Technologies, Nanyang Technological University, 637371 Singapore

[‡]OPTIMUS, Photonics Centre of Excellence, School of Electrical and Electronic Engineering, Nanyang Technological University, 639798 Singapore

[§]Optoelectronics Research Centre and Centre for Photonic Metamaterials, University of Southampton, SO17 1BJ, UK

*Corresponding author: (A.M.D.) dubrovkin@ntu.edu.sg; (Q.J.W.) qjwang@ntu.edu.sg.

S1. SiO_2 roughness and near-field images of the graphene flake exfoliated on Si-SiO_2 wafer, pre-processed by sonication (5 min) in NI555 stripper at the room temperature.

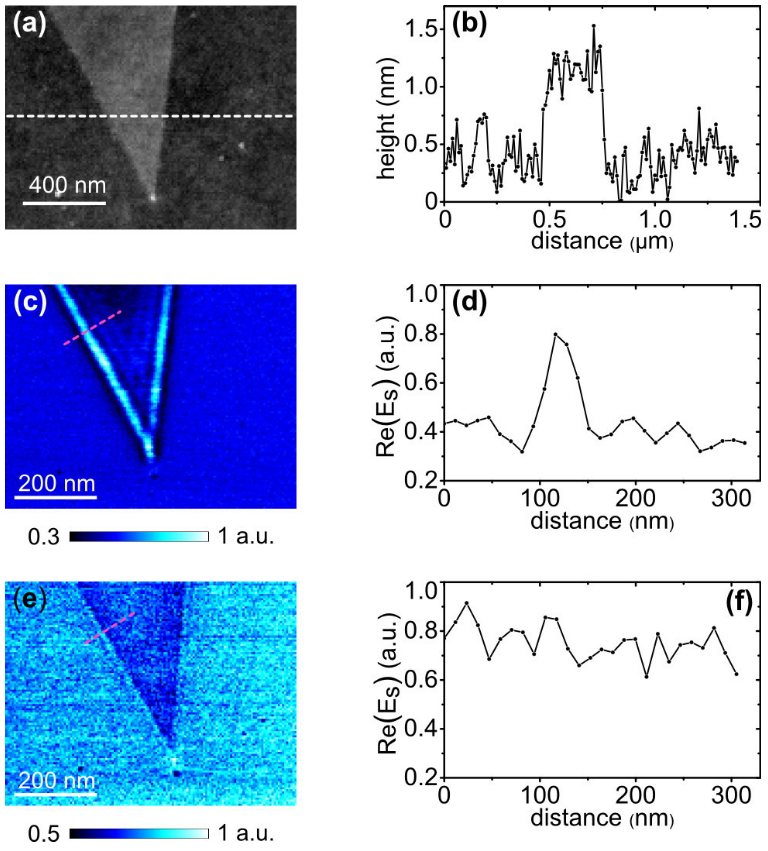

Figure S1 | (a,b). Atomic force microscopy (AFM) image of the exfoliated graphene on NI555-stripped Si-SiO_2 wafer, and a corresponding height profile along the dotted white line. (c,e). Optical near-field images of exfoliated graphene flake, recorded at λ_2 = 11.2 μm and λ_1 = 10 μm, respectively. (d,f). Corresponding cross-sections along dotted pink lines in images (c,e).

S2. Raman spectroscopy data for quasi-suspended graphene.

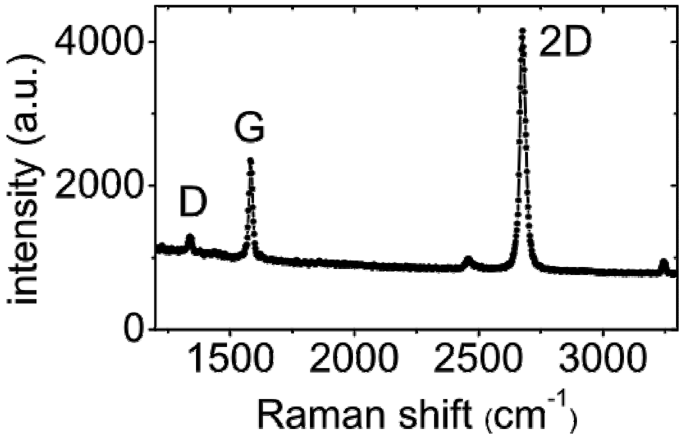

Figure S2 | Raman spectrum, measured for the graphene flake in Figure 2a.

S3. Roughness analysis of nanostructured spacer (NS), and graphene on NS.

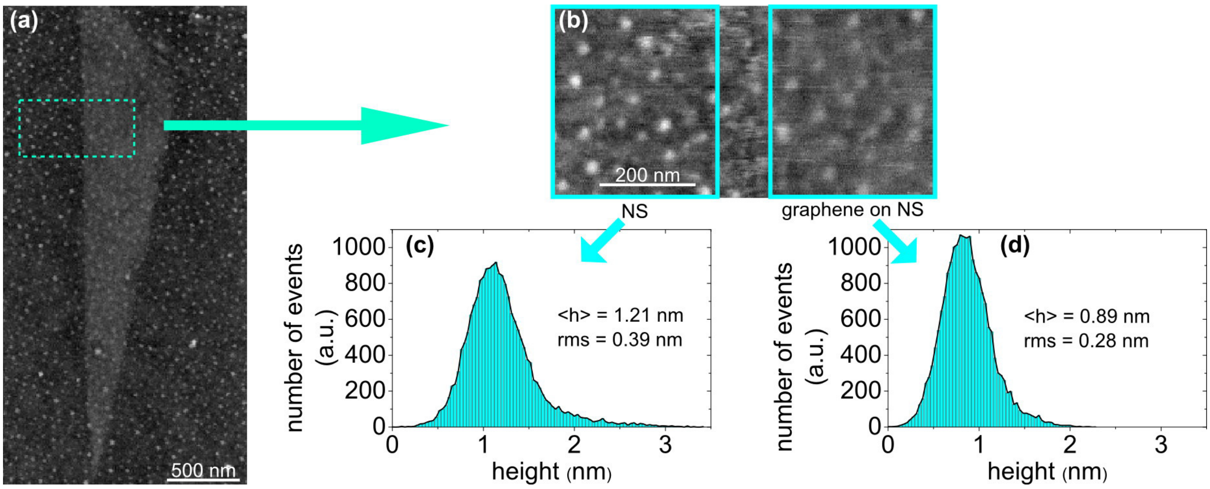

Figure S3 | (a) Reprint of the Fig. 2a. (b) Zoomed-in AFM topography of the area inside the dotted rectangle in image (a). (c,d). Corresponding relative height histograms of the data in cyan squares in image (b), which correspond to NS (left) and graphene on NS (right).

We observe a lower surface roughness (rms, root mean square) and average relative height (<h>), for graphene on NS compare to bare NS, as demonstrated in the data in Fig. S3b-d.

S4. Additional near-field and topographic images of the graphene on NS-SiO_2 at $\lambda_1 = 10$ μm.

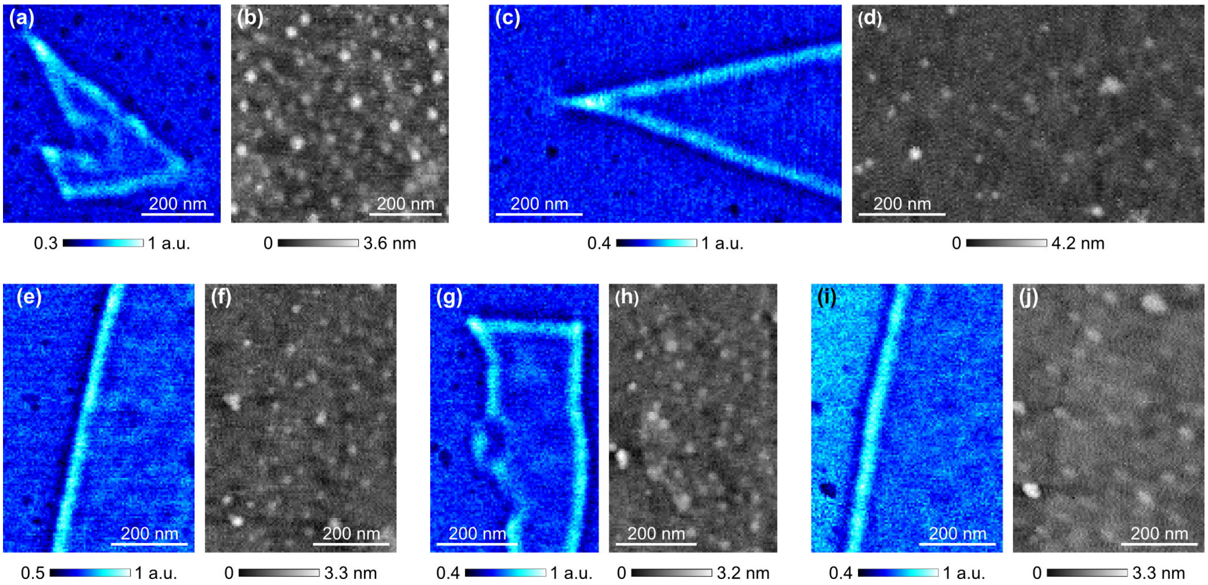

Figure S4 | (a,c,e,g,i) Optical near-field images of exfoliated graphene flakes, recorded at $\lambda_1 = 10$ μm. (b,d,f,h,j) AFM scans corresponding to images (a,c,e,g,i).

S5. Near-field image of the graphene on NS-SiO_2 at $\lambda_2 = 11.2$ µm.

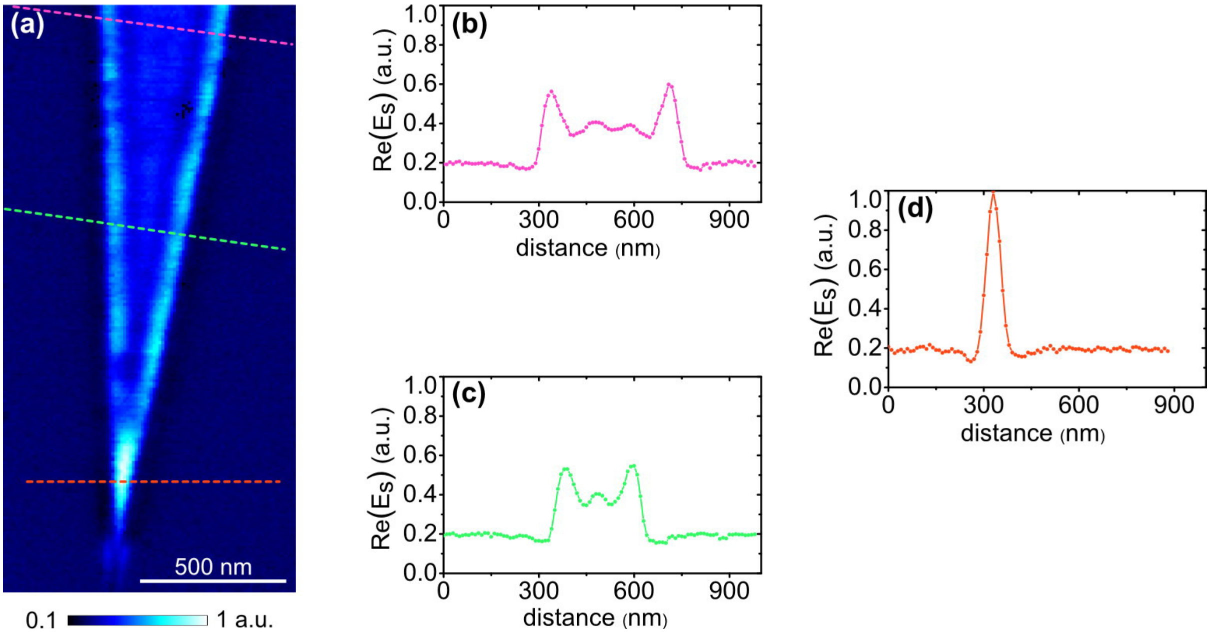

Figure S5 | (a). Optical near-field image of the exfoliated graphene flake (presented in Fig. 3 of the main text), recorded at λ_2 = 11.2 µm. (b-d). Corresponding cross-sections along dotted lines in the image (a).
